# Cellular Reprogramming Using Defined Factors and MicroRNAs

**DOI:** 10.1155/2016/7530942

**Published:** 2016-06-12

**Authors:** Takanori Eguchi, Takuo Kuboki

**Affiliations:** ^1^Division of Molecular and Cell Biology, Department of Radiation Oncology, Beth Israel Deaconess Medical Center, Harvard Medical School, 3 Blackfan Circle, Center for Life Science 6, Boston, MA 02115, USA; ^2^Department of Dental Pharmacology, Okayama University Graduate School of Medicine, Dentistry and Pharmaceutical Sciences, 2-5-1 Shikata-cho, Okayama 700-8525, Japan; ^3^Advanced Research Center for Oral and Craniofacial Sciences, Okayama University Dental School/Graduate School of Medicine, Dentistry and Pharmaceutical Sciences, Okayama 700-8525, Japan; ^4^Department of Oral Rehabilitation and Regenerative Medicine, Okayama University Graduate School of Medicine, Dentistry and Pharmaceutical Sciences, 2-5-1 Shikata-cho, Okayama 700-8525, Japan

## Abstract

Development of human bodies, organs, and tissues contains numerous steps of cellular differentiation including an initial zygote, embryonic stem (ES) cells, three germ layers, and multiple expertized lineages of cells. Induced pluripotent stem (iPS) cells have been recently developed using defined reprogramming factors such as Nanog, Klf5, Oct3/4 (Pou5f1), Sox2, and Myc. This outstanding innovation is largely changing life science and medicine. Methods of direct reprogramming of cells into myocytes, neurons, chondrocytes, and osteoblasts have been further developed using modified combination of factors such as N-myc, L-myc, Sox9, and microRNAs in defined cell/tissue culture conditions. Mesenchymal stem cells (MSCs) and dental pulp stem cells (DPSCs) are also emerging multipotent stem cells with particular microRNA expression signatures. It was shown that miRNA-720 had a role in cellular reprogramming through targeting the pluripotency factor Nanog and induction of DNA methyltransferases (DNMTs). This review reports histories, topics, and idea of cellular reprogramming.

## 1. Introduction

A term of cellular “reprogramming” has been major after the development of induced pluripotent stem (iPS) cells [1]. For the development of iPSCs, Dr. Shinya Yamanaka was awarded Nobel prize in physiology and medicine in 2012. The iPS cells are embryonic stem (ES) cells-like pluripotent cells induced using defined factors. The definition of “reprogramming” in the narrow sense is like artificial dedifferentiation (reprogram) of cells such as skin cells into ES cells-like pluripotent stem cells. Mesenchymal stem cells (MSCs), haematopoietic stem cells (HSCs), or neuronal stem cells (NSCs) are also multipotent stem cells, which are intermediate cells between more matured cells and pluripotent stem cells. These intermediate stem cells have been also investigated in reprogramming studies. More recently, a new concept termed “direct reprogramming” has been developed. Direct reprogramming is reprogramming of cells such as skin cells into another type of differentiated cells in another lineage.

## 2. Stem Cells, Germ Layers, and Tissue Development 

In order to understand cellular reprogramming, we need some basic knowledge regarding tissue development. An embryo is a multicellular diploid eukaryote in its earliest stage of development, from the time of fertilization through sexual reproduction until birth, hatch, or germination. ES cells are pluripotent stem cells derived from the inner cell mass of a blastocyst, an early-stage preimplantation embryo. In a beginning step of embryonic development from ES cells and the blastocyst, three germ layers are generated, ectoderm, mesoderm, and endoderm.

### 2.1. Ectoderm

Ectoderm emerges and originates from the outer layer of germ cells. The word ectoderm comes from the Greek ektos, meaning outside, and derma, meaning skin. The ectoderm differentiates to form the nervous system (spine, peripheral nerves, and brain) and tooth enamel via ameloblasts and epidermis (the outer part of integument). Ectoderm also forms the lining of the mouth (oral mucosa), anus, nostrils, sweat glands, hair, and nails. In vertebrates, the ectoderm has three parts, external ectoderm also known as surface ectoderm, the neural crest, and neural tube. The latter two are known as neuroectoderm as described below. Established ectodermal markers are *β*-III-tubulin and Otx2. Sasai et al. reported that ectodermal factor XFDL156 (Zfp12, Zfp74) restricts mesodermal differentiation by inhibiting p53 that is required for mesodermal differentiation [2].

### 2.2. Neuroectoderm, Neurulation, and Neural Crest

Formation process of the neural tube, neural crest cells, and the epidermis is called neurulation. The neural tube cells give rise to the central nervous system (CNS). Neural crest cells give rise to the peripheral and enteric nervous system, melanocytes, facial cartilage, and the dentin of teeth. The epidermal cell region gives rise to the epidermis, hair, nails, sebaceous glands, olfactory, and mouth epithelium as well as eyes. All of the organs that arise from the ectoderm originate from two adjacent tissue layers, the epithelium and the mesenchyme. Organogenesis of the ectoderm is mediated by signals such as FGF, TGF*β*, Wnt, and the hedgehog family. FGF-9, which is expressed in epidermis but not in the mesenchyme, is a key factor in the initiation of tooth germ development. FGF-10 helps to stimulate epithelial cell (ameloblast) proliferation, in order to make larger tooth germs. Mammalian teeth develop from oral ectoderm and neural crest ectoderm derived from mesenchyme. There are over 170 subtypes of ectodermal dysplasia.

### 2.3. Odontogenesis (Tooth Development)

Tooth germ is an aggregation of cells that eventually forms a tooth. These cells are derived from the ectoderm of the first pharyngeal arch and the ectomesenchyme of the neural crest [3, 4]. The tooth germ is organized into three parts, the enamel organ, the dental papilla, and the dental sac (or dental follicle). The cells in the enamel organ give rise to ameloblasts, which produce enamel. The location where the outer enamel epithelium and inner enamel epithelium join is called the cervical loop. The growth of cervical loop cells into the deeper tissues forms Hertwig Epithelial Root Sheath, which determines the root shape of the tooth. During the development, there are strong similarities between keratinization and amelogenesis [5, 6]. Keratin is also present in epithelial cells of tooth germ. A thin film of keratin is present on erupted tooth so called Nasmyth's membrane or enamel cuticle. The dental papilla contains cells that develop into odontoblasts, which are dentin-forming cells. Mesenchymal cells within the dental papilla are responsible for the formation of tooth pulp. The dental sac (or dental follicle) gives rise to cementoblasts, osteoblasts, and fibroblasts. Cementoblasts form the cementum of a tooth. Osteoblasts give rise to the alveolar bone surrounding the roots of teeth. Fibroblasts are involved in developing periodontal ligaments, which connect tooth cementum to the alveolar bone.

Tooth have a bone-like structure and nature, whose outside is armored by cortical calcified enamel layer. Intermediate ivory-like dentin layer of tooth contains dendrite-like process of odontoblasts. Inside of tooth is called dental pulp, which has bone-marrow-like space and contains nerves, vasculatures, haematopoietic cells, and matrix-producing cells, odontoblasts. The odontoblasts are cells lining on the surface of dentin matrix from the pulp inside and extending dendrite-like process through the dentinal tube. These structures and functions of odontoblasts are similar to osteoblasts, which are lining on the surface of bone matrix from the bone marrow. As odontoblasts are extending dendrite-like process, osteocytes are extending dendrites through the bone canaliculi. Both odontoblasts and osteocytes locate close to nerves and are the sensors as well. Such bone-marrow-like feature of dental pulp “tooth marrow” has given us an idea of dental pulp stem cells (DPSCs) or tooth marrow stromal cells (TMSCs) that may contain multiple stem cells including MSCs, HSCs, and NSCs [7–11]. Stem cells from human exfoliated deciduous teeth (SHEDs) have been shown to be useful as therapeutic tools [12].

### 2.4. Mesoderm

Mesoderm has been known as a resource of muscle (including smooth, cardiac, skeletal, tongue, mastication, and facial expressions), bone, cartilage, blood vessels, blood cells, urogenital structures such as kidney, gonads, and their associated ducts. A group of cells in ectoderm transforms via epithelial-mesenchymal transition (EMT) migrates between ectoderm and endoderm and then forms mesoderm. Mesoderm has the capacity to induce the growth of other structures, such as the neural plate, the precursor to the nervous system. Mesoderm is formed through a process called gastrulation. There are three components, the paraxial mesoderm, the intermediate mesoderm and the lateral plate mesoderm. The paraxial mesoderm gives rise to mesenchyme of the head and organizes into somites that give rise to muscle tissue, cartilage and bone, and subcutaneous tissue of skins. Signals for somite differentiation are derived from structures surrounding mesoderm, such as notochord, neural tube, and epidermis. Established markers of mesoderm are *α*-smooth muscle actin (*α*-SMA) and brachyury.

Muscle satellite cells (myosatellite cells) have been shown as precursors of mature skeletal muscle cells [13]. The muscle satellite cells have a potential to provide additional myonuclei to their parental muscle fiber or return to a quiescent state [14]. Tendon and ligament have been known to be mesenchymal lineage as well.

### 2.5. Bone Marrow Stromal Cells and Mesenchymal Stem Cells

Bone marrow stromal cells (BMSCs) have been shown to contain mesenchymal stem cells (MSCs) that have abilities to differentiate to multiple lineages such as osteoblasts, chondrocytes, and adipocytes. Osteoblasts are lining on the surface of and producing bone matrix, while osteocytes, the further differentiated cells of osteoblasts, are embedded in the lacuna in the calcified matrix, extending communicative dendrites into canaliculi for mechanotransduction [15]. Defined transcription factors can induce differentiation of MSCs into expertized lineages, for example, Sox9 in initial chondrogenesis [16], Runx2 in osteoblast precursor and chondrocyte hypertrophy [17], Osterix/Sp7 in later osteogenesis [18, 19], PPAR*γ* in adipogenesis [20], and MyoD in myogenesis [21].

### 2.6. Endothelial Cells, Haematopoietic Stem Cells, and Blood Cells

Haematopoietic stem cells (HSCs) and cardiovascular system have been known to be differentiated from mesoderm. Whether blood cells arise from mesodermal cells, mesenchymal progenitors, bipotent endothelial-haematopoietic precursors, or haemogenic endothelial cells had remained controversial, but haemangioblasts have been known to differentiate to endothelial cells as well as to blood cells. Lancrin et al. showed that the haemangioblast generates haematopoietic cells through a haemogenic endothelium stage [22]. Eilken et al. showed that using new imaging and cell-tracking methods, embryonic endothelial cells could be haemogenic [23]. Boisset et al. showed that using* in vivo* imaging, the dynamic* de novo *emergence of phenotypically defined HSCs, which were Sca1(+), c-kit(+), and CD41(+), directly from ventral aortic haemogenic endothelial cells [24]. Bertrand et al. (2010) showed that HSCs derive directly from aortic endothelium during development [25]. Chen et al. showed that Runx1 is required for the endothelial to haematopoietic cell transition but not thereafter [26]. Kissa and Herbomel showed that blood stem cells emerge from aortic endothelium by an endothelial-haematopoietic transition [27].

Blood haematopoietic cells have been known to be derived from mesoderm and located in the red bone marrow. HSCs are the stem cells that give rise to all the other blood cells through the process of haematopoiesis [28]. The HSCs give rise to the myeloid and lymphoid lineages of blood cells. Myeloid cells include monocytes, macrophages (M1 and M2), neutrophils, basophils, eosinophils, erythrocytes, dendritic cells (DCs), megakaryocytes, or its products platelets. Bone-resolving multinucleated osteoclasts are one of the mature cells derived from monocytes that differentiate to mononucleic osteoclast precursor leading to osteoclastic cell fusion. Lymphoid cells include T cells (T helper 1 cells, T helper 2 cells, and T helper 17 cells, also known as Th1, Th2, and Th17 cells, etc.), B cells, and natural killer (NK) cells. HSCs constitute 1 : 10,000 of cells in the myeloid tissue in bone marrow. Artery, vein, nerves, and lymphatic vessels are entering and extending in bone. As part of the lymphatic system, lymph vessels are complementary to the cardiovascular system.

### 2.7. Endoderm Including Definitive Endoderm and Mesoendoderm

Endoderm is an origin of multiple organs such as pancreas (Pdx1, Ngn3, Ins are the markers), liver, gut, intestine, lung, thyroid gland and thymus, urinary system such as urinary bladder, and urethra. The endoderm consists of flattened cells, which subsequently become columnar. It forms the epithelial lining of multiple systems. The embryonic endoderm develops into the interior lining of the digestive tube and respiratory tube.

Endoderm gives rise to definitive endoderm (DE) and visceral endoderm. Interestingly, a part of DE forms mesendoderm that is originated from common precursor cells derived from mesoderm and endoderm. The DE gives rise to the gastrointestinal organs, such as stomach, pancreas, liver, and intestine. Recent studies have identified several germ layer-specific markers of the early DE. Sox17 is a DE-specific marker [29]. CXCR4 (C-X-C chemokine receptor type 4), which is expressed in the mesoderm as well as in the DE and is used in combination with E-cadherin, which is also expressed in ES cells and ectoderm, for the prospective isolation of embryonic or ES cell-derived DE cells [30]. Dr. Kume's group identified DAF1 (decay accelerating factor)/CD55 and Cerberus1 (Cer1) as novel DE markers [31, 32]. Shiraki et al. reported differentiation and characterization of ES cells into three germ layers using mesoderm-derived feeder M15 cells [33, 34]. In this study, stimulation with a combination of activin and bFGF induced ES cell differentiation to mesendoderm, endoderm, and pancreatic precursor cells; stimulation with Bmp7 induced differentiation to mesoderm; the addition of p38 MAPK inhibitor SB203580 induced differentiation to neuroectoderm. Further long-term culture enabled neuroectodermal differentiation to neuron, astrocytes, and oligodendrocytes and mesodermal differentiation to osteoblasts and adipocytes. It was also shown that stimulation of endoderm with a combination of HGF and dexamethasone induced differentiation to hepatocytes.

Characterization of stem cells in the development of bodies, organs, and tissues has been our scientific interests while the multipotency of differentiation, proliferation, and self-renewal have been emerging in clinical applications.

## 3. Induced Pluripotent Stem Cells

In 2006, Takahashi and Yamanaka reported induction of pluripotent stem (iPS) cells from mouse adult fibroblast cultures by defined factors [1]. Before this study, numerous transcription factors (TFs) had been shown to be critical to induce or maintain certain cellular differentiation stages including ES cells. Dr. Yamanaka's group aimed to know which factors were required for induction of ES cells-like pluripotency. More than 20 transcription factors and their combinations were examined using retrovirus-mediated transduction in skin fibroblasts. Nanog is one of the TFs that are expressed in ES cells, and Nanog promoter-driven green fluorescence protein (GFP) reporter was used as a marker for the screening of the TFs. Another marker is the colony formation ability of pluripotent stem cells. Utilizing these significant features, Dr. Yamanaka's group finally discovered that ES cells-like colonies were induced from skin fibroblasts using combination of four defined factors, Klf4, Oct-3/4 (Pou5f1), Sox2, and c-Myc. The ES cells had been known to have an ability to form teratoma upon injection to experimental animals. Both ES and iPS cells generated from skin fibroblasts indeed formed teratoma upon subcutaneous injection to mice. Finally, the ESC-like iPSCs were shown to be able to give rise to embryos. These results were enough to say that the cells were pluripotent. The iPS cells were next generated from human dermal fibroblasts [35]. So far, established markers of ES and iPS cells are SSEA-3, SSEA-4, TRA-1-60, TRA-1-81, Oct-4 (Pou5f1), Nanog, Sox2, Klf4, MycN, Lin28, Cripto, Fbx15, Dnmt3b, Fgf4, Gdf3, Rex1, miR-200c, miR-302 family, miR-369-3p, and miR-369-5p.

### 3.1. Improvement of Methods in Generation of iPS Cells

After this breakthrough of iPS cells, researchers have made the effort to reduce risks of oncogenesis, which could be induced during the reprogramming. The risk included the usage of viral vectors that could be integrated into genomic DNA and the usage of an oncogene c-myc. Replacement of the viral vectors to plasmid vectors enabled to generate iPS cells while induction efficiency was reduced [36]. One clue was that the oncogenic c-myc is a member of Myc family that includes other two members N-myc and L-myc. Dr. Yamanaka's group replaced the c-myc to N-myc and L-myc [37]. Indeed, iPS cells were generated using N-myc instead of c-myc.

**Figure 1:**
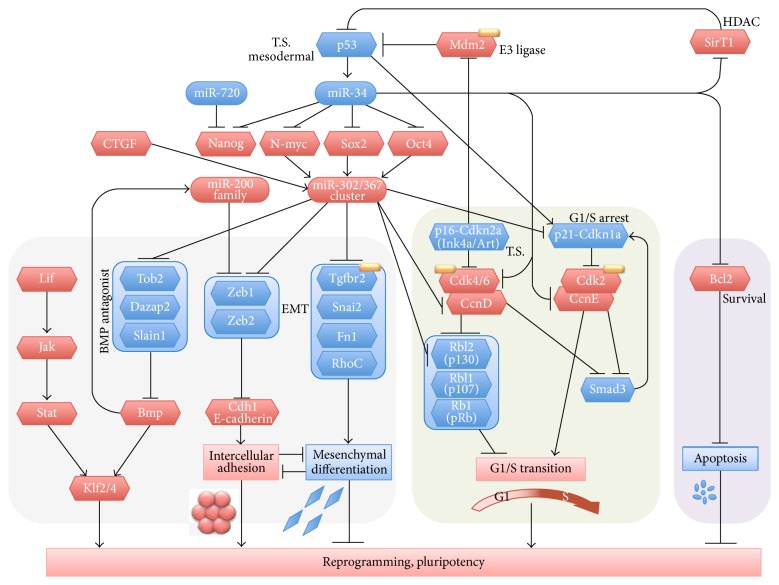
Summary of published intermolecular interactions featuring pluripotency-associated miRNAs and their targets. Factors positive to induction of pluripotency was shown in red. Inhibitory factors to induction of pluripotency was shown in blue. Epithelial-mesenchymal transition (EMT), cell cycle, and cell death and survival were featured. Molecularly targeted drugs are shown with drug marks. T.S., tumor suppressor.

It had been known that p53–p21 pathway served as a barrier in tumorigenicity (Figure 1). Dr. Yamanaka's group demonstrated that the p53–p21 pathway was also a barrier in the generation of iPS cells [38]. The discovery of iPS cells and the recovery from the tumorigenic risk have made the cells more feasible toward clinical applications.

### 3.2. Clinical Application of Pluripotent Stem Cells

The application of ES and iPS cells has been thought for regenerative medicine. The cells or tissues generated by the reprogramming might be useful for transplantation therapies. Hotta and Yamanaka reviewed clinical applications of pluripotent stem cells in their recent publication entitled “From Genomics to Gene Therapy: Induced Pluripotent Stem Cells Meet Genome Editing” [39]. The world's first phase I clinical trial using hES cells was aimed to treat patients with spinal cord injuries. In October 2010, oligodendrocyte progenitor cells derived from H1 human ES cells were injected into the site of spinal cord damage. Unfortunately, the trial was terminated in 2013 after the biotech company Geron decided to withdraw from the stem cell business. The second clinical trial using hES cells investigated Stargardt's macular dystrophy (SMD) and advanced dry age-related macular degeneration (Dry AMD) by using retinal pigment epithelium (RPE) cells derived from a human ES cell line MA09. The first patients for each trial was treated in 2011. As a phase I study to test feasibility and safety, there were no signs of tumorigenicity of apparent rejection [40]. The third example of an ES cell-related clinical trial is for type I diabetes. Pancreatic precursor cells (called PEC-01 cells) from CyT203 human ES cells [41] were encapsulated into a device, Encaptra, and transplanted into a patient in 2014 as a phase I/II clinical trial. In all trials, transplantation of human ES cells-derived products was allogenic and has been conducted without matching HLA types. Therefore, the current applications are mainly limited to immune privileged tissues, such as eyes and spinal cord. iPS cells have been investigated in an autologous transplantation setting. In September 2014, the first transplantation of RPE cells derived from iPS cells [42] for Wet AMD was conducted by Dr. Takahashi's group at RIKEN CDB in Japan.

A potential application of iPS cells has been advocated that this concept and methods can be useful for generation of disease model, in which cells can be reprogrammed from patient cells. Such patient-derived iPS cells can be reasonably used for drug screening. Yahata et al. (2011) reported antiamyloid *β* (A*β*) drug screening platform using human-iPS cells-derived neurons for the treatment of Alzheimer's disease [43]. Dr. Takahashi's group (2014) reported integration-free iPS cells derived from retinitis pigmentosa patient for disease modeling [44]. Suzuki et al. reported that pluripotent cell models of Fanconi anemia identify the early pathological defect in human haemoangiogenic progenitors [45].

Genetic linkage analysis and recent genome-wide association studies (GWAS) meta-analysis have identified human genetic variation/mutations associated with diverse diseases and physical characteristics [46]. Genetic variations/mutations with diseases could be corrected to normal or lower risk variation with genome editing technologies using CRISPR/Cas9 that requires noncoding guide RNA (gRNA) [47]. Indeed, Kazuki et al. reported complete genetic correction of iPS cells from Duchenne muscular dystrophy (DMD) [48]. Morishima et al. reported that genetic correction of HAX1 in iPS cells from a patient with severe congenital neutropenia improves defective granulopoiesis [49]. Further application of iPS cells in combination with genome editing therapy and for generation of disease model in terms of drug discovery is ongoing.

## 4. MicroRNAs Involving Pluripotent Stem Cells

MicroRNAs (miRNAs) are short noncoding RNAs that target long RNAs leading to inhibition of translation and/or promotion of mRNA degradation. miRNAs are generated from miRNA cluster or intron of coding RNA. These resources of miRNA are transcripts of genomic DNA. miRNA also have been thought to be useful as biomarkers, tools for cellular reprogramming, and therapeutic targets. One miRNA species targets multiple long RNA leading to translational inhibition of multiple proteins. Nevertheless, several challenges to control cellular stemness and differentiation by miRNA have been done.

### 4.1. MicroRNA-302/367 Cluster Targets Multiple Factors Involving Epithelial-Mesenchymal and G1-S Transitions

miRNA-induced pluripotent stem cells (mirPSC) were firstly reported using human skin cancer cells. Lin et al. reported that miR-302 reprogrammed human skin cancer cells into a pluripotent ESC-like state [50]. This group firstly reported that the miR-302 family members (miR-302s) were expressed most abundantly in slowly growing human ES cells and “quickly” decreased after cell differentiation and proliferation. Therefore, they hypothesized that miR-302s could be one of the key factors essential for maintenance of ESC-renewal and pluripotency. The miR-302-transfected cells expressed key ES cell markers such as Oct3/4, SSEA-3/4, Sox2, and Nanog but also had a highly demethylated genome similar to a reprogrammed zygotic genome. Microarray analysis further revealed that genome-wide gene expression patterns between the mirPSCs and human ES cells H1 and H9 shared over 86% similarity. Using molecular guidance* in vitro*, this mirPSC could differentiate into distinct tissue cell types such as neurons, chondrocytes, fibroblasts and spermatogonia-like cells. Based on these findings, this group concluded that miR-302s function to reprogram cancer cells into an ES-like pluripotent state but also to maintain the stem cell state under a feeder-free cultural condition.

Thereafter, Barroso-del Jesus et al. (2009) released perspectives regarding the miR-302/367 cluster as a potential stemness regulator in ES cells [51]. Later miRNA profiling studies reproduced that miR-302 was essentially expressed specifically in ES and iPS cells and lost upon differentiation and proliferation. One such profiling was carried out by Wilson et al., who reported microRNA profiling of human-iPS cells termed microRNAomes [52]. This group confirmed that the presence of a signature group of miRNAs that is upregulated in both iPS and hES cells, such as the miR-302/367 and miR-17/92 clusters. Another miRNA profiling was carried out by Stadler et al., who reported the characterization of microRNAs involved in a state of ES cells [53]. This group showed that defined hES cells-enriched miRNA groups (miR-302, miR-17, miR-515 families, and the miR-371-373 cluster) were downregulated “rapidly” in response to differentiation.

One arising question was what factors were targeted by miR-302. The first report of targets of miR-302 in ES and iPS cells was regarding cell cycle regulators. Card et al. showed that Oct4/Sox2-regulated miR-302 targeted mRNA encoding cyclin D1 in hES cells [54] (Figure 1). Lin et al. reported that maintaining slowly proliferating adhesive stem cells will require inhibition of cell cycle by miR-302/367 that targets cyclin D1 and cyclin-dependent kinases 2/4/6 (Cdk2, Cdk4 and Cdk6) [55]. Nevertheless, cell cycle must be rotated for mitosis of iPS and ES cells. A tumor suppressor p53 had been shown to upregulate p21·Cdkn1a (Cdk inhibitor 1a). p21/Cdkn1 had been known to inhibit Cdk2/cyclin E (CcnE) complex leading to G1/S arrest. Wang et al. reported that ES cell-specific microRNAs regulated G1-S transition and promote rapid proliferation [56]. In this study, G1-S arrest factors including p21/Cdkn1a, p130/Rbl2, and Lats2 were shown to be targeted by 5′-UAAAGUGC…-containing or AAAGUGCU…-containing microRNAs such as miR-291, miR-294, miR-295, and miR-302d. This work explained how the miR-302 overcame the G1-S arrest at the G1-S transition. Lin et al. (2011) reported that miR-302 targeted cosuppression of four “epigenetic” regulators, lysine demethylase KDM1 (also known as LSD1/AOF2), AOF1, p66/MECP1, and MECP2 [57].

Targeting TGF*β* signaling by miR-302 may reprogram cells toward generation of iPS and mirPS cells through induction of mesenchymal-epithelial transition (MET), the acquisition of intercellular adhesion. Pluripotent stem cells have characters to form colonies along with acquirement of intercellular adhesion. Intercellular adhesion is known largely to be lost during EMT in tissue development. The most significant inducer of EMT is TGF*β*, and loss of TGF*β* signaling can induce epithelial phenotypes with intercellular adhesion. Thus, the generation of iPS cells may require MET along with the acquisition of intercellular adhesion.

Sequencing of RNA transcripts revealed that a pre-miRNA cluster encoded five miRNAs including miR-302a, -302b, -302c, -302d (miR-302s), and miR-367, termed miR-302/367 cluster. Liao et al. reported that the miR-302/367 cluster enhanced somatic cell reprogramming (SCR) by accelerating an MET through targeting TGF*β* type II receptor (TGFbR2) and increased E-cadherin expression [58]. BMP signaling had been known as being required for maintenance of ES cells. Lipchina et al. reported that miR-302/367 cluster promotes BMP signaling by targeting BMP inhibitors TOB2, DAZAP2, and SLAIN1 [59] (Figure 1). Li et al. reported that not only miR-302 but also miR-93 targets mRNA encoding TGFbR2 to enhance generation of iPS cells [60]. Anokye-Danso et al. reported miRNA-302/367-mediated reprogramming of mouse and human somatic cells to pluripotency [61]. This work showed an extremely higher efficiency of ES cell-like colony formation with ES cell-like morphology and expression of markers using miR-302/367 cluster compared to OSKM-iPS. In this study, the number of colonies with ES cell-like morphology per 100,000 cells was 10396 cells using miR-302/367 and only 3 with OSKM in this work. However, one says that, for years, this work has not been reproduced at all in any other groups or extensively used (given that the efficiency in the paper is strikingly high). Poleganov et al. reported that human fibroblasts and “blood-derived endothelial progenitor” cells were efficiently reprogrammed by transduction of nonmodified miR-302/367 cluster in a single vector leading to immune evasion [62]. These works using miR-302/367 showed that blocking mesenchymal TGF*β* signaling and maintenance of BMP signaling were required for generation and maintenance of iPS cells. Switching from BMP to TGF*β* signaling may enable cells to differentiate to mesenchymal lineage/phenotype.

Faherty et al. reported that CCN2/CTGF increased miR-302s expression level [63] (Figure 1). CCN2 has been known to associate with diverse extracellular proteins including growth factors, cell surface molecules such as receptors and integrin, and extracellular matrix proteins leading to modulation of these partners [64, 65]. Thus, CCN2/CTGF may be useful as a growth factor supporting cellular reprogramming through induction of miR-302.

### 4.2. Combination of miR-200c Plus miR-302 s and miR-369 s Family

miR-200 family has been shown to repress EMT through targeting Zeb1/Tcf8/deltaEF1, a master transcription factor that represses E-cadherin gene (CDH1). Miyoshi et al. reported “reprogramming of mouse and human cells to pluripotency using mature microRNAs” [66, 67]. This group used a combination of miR-200c plus miR-302 s and miR-369 s family of miRNAs without using OSKM factors. Miyoshi et al. showed similarity and difference between mirPS and ES cells in gene expression levels of markers such as Nanog, Oct4, Cripto, Dppa5, Eras, Fbx15, and miRNAs, on which they focused. Leonardo et al. featured these successful mature miRNA-driven reprogramming in a review entitled “the functions of microRNAs in pluripotency and reprogramming” [68].

### 4.3. miR-34a Induced by Tumor Suppressor p53 Targets Multiple Pluripotency Factors, Cell Cycle Genes, and Antiapoptotic Bcl2

The p53-p21 pathway had been known as a tumor suppressor to G1-S arrest axis (Figure 1). Hong et al. showed suppression of iPS cell generation by the p53–p21 pathway [38]. In this study, iPS cell generation efficiency was largely increased by knockdown and knockout of p53. In other words, loss of the tumor suppressor p53 may induce pluripotency in carcinogenesis. In the p53-null background, iPS cells were generated from terminally differentiated T lymphocytes.

Interestingly, in 2007, four groups reported that microRNA-34 family members were a novel transcriptional target of tumor suppressor p53 in human colon cancer cells or in mouse embryonic fibroblasts (MEFs) [69–74]. In these works, it was shown that miR-34 family miRNAs mediated cellular senescence (growth arrest) in human colon cancer cells or MEFs and targeted a program of genes promoting cell cycle progression such as E2F, Cdk4, CcnE2, and Met (Figure 1). Referring to these works mentioned above, Choi et al. showed that miR-34 provided a barrier for somatic cell reprogramming [75]. This work showed OSMK-triggered, p53-dependent induction of miR-34 s in MEFs. This work also showed that miR-34a deficiency in mice significantly increased reprogramming efficiency and kinetics, while miR-34a and p21, the downstream factors of p53, were cooperatively barrier of somatic reprogramming. It was shown that suppression of reprogramming by miR-34a was due to repression of pluripotency genes, Nanog, Sox2, and N-myc. Cole et al. showed miR-34a as a candidate tumor suppressor gene in neuroblastoma through targeting antiapoptotic Bcl2 and pluripotency factor N-myc [76] (Figure 1). Removing such barrier factors are a reasonable strategy for the establishment of barrier-free reprogramming in terms of efficient and reproducible reprogramming.

## 5. Direct Reprogramming

The reprogramming of cells to iPS cells gives the cells possessing abilities of pluripotency and self-renewal. Another idea has been challenged that cells in a mature lineage could be directly transdifferentiated into the other lineages using defined factors termed as “direct reprogramming.” Since EMT and MET have been shown during the development of body and cancer, the induced transition from one lineage to the other is realistic. Moreover, the short cut direct reprogramming without iPSC generation may be more reasonable, efficient, and beneficial for clinical application.

### 5.1. Induced Chondrocyte-Like Cells (iChon)

Dr. Nobuyuki Tsumaki's group have reported the direct reprogramming of human dermal fibroblasts to chondrocytes using defined factors [77–84]. A key idea was replacing Sox2 one of the indispensable factors for the iPSC generation to its brother Sox9 that is a master transcription factor for chondrogenesis. This group has established a model of type II collagenopathy skeletal dysplasia using direct conversion and iPS cells [85, 86].

### 5.2. Induced Neuronal Stem Cells (iNSC) and Induced Neurons (iN)

The combination of miR-124 and two TFs MYT1L and BRN2 was sufficient for direct reprogramming of postnatal and adult human primary dermal fibroblasts to functional neurons [87]. Han et al. reported direct reprogramming of fibroblasts into NSCs using defined factors including SMOK factor plus TCF3/E47 [88]. The requirement of *β*-catenin signaling for induced NSC (iNSC) was shown in this study, because the TCF3 is a recipient TF of the Wnt/*β*-catenin signaling.

Not addition but subtraction of defined factors has challenged for the iNSC generation. Ring et al. reported direct reprogramming of mouse and human fibroblasts into multipotent NSCs with only a single factor Sox2 in an “NSC permissive culture condition” [89]. Wapinski et al. reported a hierarchical mechanism in the direct conversion of fibroblasts into induced neurons (iN) cells mediated by Ascl1, Brn2, and Myt1L transcription factors [90].

Guo et al. reported* in vivo* direct reprogramming of reactive glial cells into functional neurons after brain injury and in an Alzheimer's disease model [91]. In the study, retroviral expression of a single neural TF, NeuroD1 was used for direct reprogramming of reactive glial cells into functional neurons* in vivo* in the cortex of stab-injured or Alzheimer's disease model mice.

### 5.3. Induced Cardiomyocyte-Like Cells (iCM)

Fu et al. reported direct reprogramming of human fibroblasts toward cardiomyocyte-like state [92]. This group first reported that three TFs (GATA4, MEF2C, and TBX5) termed GMT directly reprogrammed nonmyocyte mouse heart cells into induced cardiomyocyte-like cells (iCMs). Then, this group showed that GMT plus ESRRG and MESP1 induced global cardiac gene expression and phenotypic shifts in human fibroblasts. In addition, myocardin, ZFPM2, and TGF*β* signaling were shown to be important for the iCM reprogramming. Jayawardena et al. reported that a combination of miR-1, -133, -208, and -499 was capable of inducing direct cellular reprogramming of fibroblasts into cardiomyocyte-like cells* in vitro* [93].

### 5.4. Induced Vascular Endothelial Cells (iVEC)

Vascular endothelial cells were generated from fibroblasts via partial-iPSC reprogramming with four TFs required for iPSC generation and subsequent transduction of SETSIP with VEGF [94]. This group showed the SETSIP translocated to nuclei upon stimulation with VEGF and bound to the VE-cadherin promoter and increased VE-cadherin expression levels and EC differentiation.

### 5.5. Induced Osteoblast-Like Cells (iOB)

Direct conversion of human fibroblasts into functional osteoblasts by defined factors was recently reported. Yamamoto et al. reported that osteogenic transcription factors, Runx2, and Osterix/Sp7, in combination with Oct4 and L-myc, drastically induced fibroblasts to produce calcified bone matrix and osteoblastic markers [95]. This group reported that the induced osteoblasts (iOBs) into bone defects contributed to bone repair in mice. In addition, iOBs did not require continuous expression of the exogenous genes to maintain their phenotypes. Mizoshiri et al. reported that L-myc in combination with either Oct3/4, Oct6, or Oct9 enabled the conversion of fibroblasts to osteoblasts [96]. Alternatively, Oct9 plus N-myc had the strongest capability of inducing osteoblastic phenotype. Prior to these iOBs studies, Song et al. reported that loss of Wnt/*β*-catenin signaling caused cell fate shift of preosteoblasts to adipocytes [97].

MicroRNAs have been used for generation of iPSC and direct reprogramming as described above. We have had an idea that miRNA produced during osteogenic differentiation from stem cells (termed OstemiR) may be biomarkers, tools, and therapeutic targets, which are beneficial for patients suffering from osteoporosis, bone fracture, genetic osteogenic disorders, arthritis, and aging. We screened such OstemiR [98] (a partial list was shown in Table 1). The OstemiR database provides gene expression signature of miRNA and mRNA that were altered in osteoblastic/osteocytic differentiation of MSCs. The combination of some OstemiR can be a tool for direct reprogramming to osteogenic lineage.

## 6. miR-720 Promotes Dental Pulp (Stem) Cell Differentiation via Targeting Pluripotent Stem Cell Factor Nanog

We reported that microRNA-720 (miR-720) controlled stem cell phenotype, proliferation, and differentiation of human dental pulp cells [99]. Dental pulp includes mesenchymal (stem) cells, peripheral nerves, blood vessels, and blood. The mesenchymal stem cells have been known to have an ability to differentiate to mature odontoblasts that produce extracellular matrix proteins. We challenged a hypothesis that dental pulp could include multipotent stem cells. Side population (SP) cells, which had been known to be enriched with stem cells having pluripotency, in dental pulp cells were separated from main population (MP) cells with fluorescence-activated cell sorting (FACS) using Hoechst blue and Hoechst red. ABCG2, Nanog, and Oct-4 were expressed at higher levels, while a level of miR-720 expression was lower in SP cells compared to MP cells. miR-720 was predicted to target Nanog, and this targeting was experimentally proven (Figure 1). Neutralization of miR-720 using anti-miR-720 was shown to repress odontoblastic differentiation.

## 7. miR-720 Promotes Dental Pulp (Stem) Cell Differentiation through Induction of DNA Methyltransferases

We showed that miR-720 positively regulated expression of DNA methyltransferases Dnmt3a and Dnmt3b as well. In addition, overexpression of Dnmt3a and Dnmt3b promoted odontoblastic differentiation of dental pulp cells and repressed Nanog expression. Thus, it was suggested that odontoblastic differentiation of dental pulp cells required miR-720 that repressed Nanog and induces Dnmt3a and Dnmt3b.

Prior to our study, roles for Dnmt3a/b in promotion of HSC differentiation had been shown. Okano et al. showed that Dnmt3a and Dnmt3b were essential for* de novo* methylation and mammalian development, using Dnmt3a and Dnmt3b deficient mice [100]. Mutations in human DNMT3B were found in immunodeficiency, centromeric instability, and facial anomalies syndrome (ICF syndrome) in this study. Challen et al. (2012) showed that Dnmt3a was essential for HSC differentiation [101]. In this study, loss of Dnmt3a in HSCs led to higher expression of HSC multipotency genes, Runx1, Gata3, and Nr4a2. Thus, it was suggested that HSCs could differentiate into B cells via Dnmt3b-dependent hypermethylation of Runx1, Gata3, Nr4a2, and Vasn genes. Challen et al. (2014) reported that Dnmt3a and Dnmt3b might promote DNA methylation in Ctnnb1, which encodes *β*-catenin, stem cell self-renewal factor, CcnD1 a G1-phase cyclin gene, PparG an adipogenic transcription factor, and VegfA and Jag1 genes [102]. The roles for Dnmt in HSC differentiation may be similar with that in DPSCs. Thus, targeting *β*-catenin, PAPR*γ*, and CcnD1 by Dnmt3a/3b is thought to promote differentiation of cells in odontoblastic and osteoblastic lineage.

These works suggested that direct reprogramming to mature odontoblasts as well as osteoblasts could be challenged using a combination of miR-720 and Dnmt3a/3b overexpression. Another idea is that dental pulp (stem) cells are a good resource of cells having potential for generation of iPS cells from dental pulp (stem) cells. Oda et al. reported highly efficient generation of iPS cells from human third molar mesenchymal stromal cells [103].

## 8. Conclusion

Improvement of concept, materials, and methods in cellular reprogramming has given huge progress in life science and medicine. Firstly, growth factors and transcription factors were defined useful for generation of iPS cells. Later studies have shown microRNAs are also useful for the generation of iPS cells. Defined factors including growth factors, TFs, and miRNAs have been shown to be useful for cellular reprogramming. The combination of the cellular reprogramming with genome editing is ongoing in life science and medicine toward benefits for patients in human society.

## Figures and Tables

**Table 1:** List of OstemiR and their potent targets. PTM, posttranslational modification factor.

	Growth factor	Receptor	Signal, PTM	Transcription factor	Epigenetic regulator	Cell cycle regulator	Others
miR-30a, miR-30d, miR-30e	Nov, Igf, Bdnf	Lrp6, LifR, Notch, Igf2R, Igf1R, Integrin a5/a4, Integrin b3	Senp5	Sox9, *Runx2*, *Smad1/2*, FoxO3, Klf9/11, Zeb2, Snai1, NcoA1, HoxB8, Lin28b	Tet1, Tet3, SirT1, Hdac5, Eed, Mbd6	Cyclin E2/T2/K, Cdk6	*HspA5/Grp78*

miR-30b, miR-30c	Spp1/Opn, Ccn2	Lrp6, LifR		Sox9, Runx2, Lin28a, hnRnpA3, Pcgf5, Zbtb41/44	SirT1, Eed		*HspA5/Grp78*

miR-21	TgfB1, Fgf1	Lrp6, TgfbR2, BmpR2, AcvR1c, LifR, ItgB8	Ski, Smad7	Sox2/5, Klf3/5/12, Msx1	Tet1		Reck, Timp3

miR-16/miR-503	Wnt3a/4, Fgf7, Ihh, VegfA, Igf1	BmpR1a, AcvR2b, InsR, Igf1R	Smad7, Smurf1, Smurf2	Sox5, Myb, MybL1, FosL1		Runx1T1, Cyclin E1/D1/D2/D3/T2/M2, Cdk5r1/6	Hspg2, Reck

miR-155	Gdf6, Fgf7	TgfbR2, AcvR2b, Lrp1b		Tcf4, Smad1/2, Klf3, Fos, Sp1/3	SirT1	Cyclin D1	SmarcAD1, claudin1

miR-541	Wnt11	Integrin *α*3		Baz1b, Sp1	Ago1		Adamts7, Timp2
